# Bifurcation analysis of multistability and hysteresis in a model of HIV infection

**DOI:** 10.18699/VJGB-23-88

**Published:** 2023-12

**Authors:** I.V. Mironov, M.Yu. Khristichenko, Yu.M. Nechepurenko, D.S. Grebennikov, G.A. Bocharov

**Affiliations:** Keldysh Institute of Applied Mathematics of the Russian Academy of Sciences, Moscow, Russia Sechenov First Moscow State Medical University of the Ministry of Health of the Russian Federation, Moscow, Russia; Keldysh Institute of Applied Mathematics of the Russian Academy of Sciences, Moscow, Russia Marchuk Institute of Numerical Mathematics of the Russian Academy of Sciences, Moscow, Russia; Keldysh Institute of Applied Mathematics of the Russian Academy of Sciences, Moscow, Russia Marchuk Institute of Numerical Mathematics of the Russian Academy of Sciences, Moscow, Russia; Sechenov First Moscow State Medical University of the Ministry of Health of the Russian Federation, Moscow, Russia Marchuk Institute of Numerical Mathematics of the Russian Academy of Sciences, Moscow, Russia; Sechenov First Moscow State Medical University of the Ministry of Health of the Russian Federation, Moscow, Russia Marchuk Institute of Numerical Mathematics of the Russian Academy of Sciences, Moscow, Russia

**Keywords:** mathematical model, HIV infection, ordinary differential equations, bifurcation analysis, stationary solutions, bistability, multistability, hysteresis, optimal control, математическая модель, ВИЧ-инфекция, обыкновенные дифференциальные уравнения, бифуркационный анализ, стационарные решения, бистабильность, мультистабильность, гистерезис, оптимальное управление

## Abstract

The infectious disease caused by human immunodeficiency virus type 1 (HIV-1) remains a serious threat to hu- man health. The current approach to HIV-1 treatment is based on the use of highly active antiretroviral therapy, which has side effects and is costly. For clinical practice, it is highly important to create functional cures that can enhance immune control of viral growth and infection of target cells with a subsequent reduction in viral load and restoration of the immune status. HIV-1 control efforts with reliance on immunotherapy remain at a conceptual stage due to the complexity of a set of processes that regulate the dynamics of infection and immune response. For this reason, it is extremely important to use methods of mathematical modeling of HIV-1 infection dynamics for theoretical analysis of possibilities of reducing the viral load by affecting the immune system without the usage of antiviral therapy. The aim of our study is to examine the existence of bi-, multistability and hysteresis properties with a meaningful mathematical model of HIV-1 infection. The model describes the most important blocks of the processes of interaction between viruses and the human body, namely, the spread of infection in productively and latently infected cells, the appearance of viral mutants and the develop- ment of the T cell immune response. Furthermore, our analysis aims to study the possibilities of transferring the clinical pattern of the disease from a more severe state to a milder one. We analyze numerically the conditions for the existence of steady states of the mathematical model of HIV-1 infection for the numerical values of model parameters correspond- ing to phenotypically different variants of the infectious disease course. To this end, original computational methods of bifurcation analysis of mathematical models formulated with systems of ordinary differential equations and delay differ- ential equations are used. The macrophage activation rate constant is considered as a bifurcation parameter. The regions in the model parameter space, in particular, for the rate of activation of innate immune cells (macrophages), in which the properties of bi-, multistability and hysteresis are expressed, have been identified, and the features characterizing transi- tion kinetics between stable equilibrium states have been explored. Overall, the results of bifurcation analysis of the HIV-1 infection model form a theoretical basis for the development of combination immune-based therapeutic approaches to HIV-1 treatment. In particular, the results of the study of the HIV-1 infection model for parameter sets corresponding to different phenotypes of disease dynamics (typical, long-term non-progressing and rapidly progressing courses) indicate that an effective functional treatment (cure) of HIV-1-infected patients requires the development of a personalized ap- proach that takes into account both the properties of the HIV-1 quasispecies population and the patient’s immune status.

## Abstract (ru)

Инфекционное заболевание, вызванное вирусами иммунодефицита человека первого типа (ВИЧ-1), остается серьезной угрозой здоровью людей. Существующий подход к лечению ВИЧ-1 основан на применении высокоактивной антиретровирусной терапии, имеющей побочные эффекты для здоровья и высокую стоимость. Для практической медицины актуальной является задача поиска методов функционального лечения, связанных с интенсификацией иммунного контроля размножения вирусов и заражения клеток-мишеней с последующим снижением уровня вирусной нагрузки и восстановления иммунного статуса. Исследования в области иммунотерапии ВИЧ-1 находятся на стадии концептуальной разработки в силу сложности совокупности процессов, регулирующих динамику инфекции и иммунного ответа. По этой причине чрезвычайно актуальным является использование методов математического моделирования динамики ВИЧ-1 инфекции для теоретического анализа возможностей снижения вирусной нагрузки путем воздействия на иммунную систему без применения антивирусной терапии. Целью исследования было изучение, во-первых, свойств би-, мультистабильности и гистерезиса на примере содержательной модели ВИЧ-1 инфекции, которая описывает важнейшие блоки процессов взаимодействия вирусов и организма человека, а именно: распространение инфекции в продуктивно и латентно зараженных клетках, появление мутантов и развитие Т-клеточного иммунного ответа, и, во-вторых, возможностей перевода клинической картины заболевания из более тяжелого состояния в более легкое. В данной работе проведен численный анализ условий существования стационарных решений математической модели ВИЧ-1 инфекции для наборов параметров, отвечающих фенотипически различным вариантам течения инфекционного заболевания. Для этого использованы разработанные авторами методы бифуркационного анализа моделей, представляющих собой системы обыкновенных дифференциальных уравнений и дифференциальных уравнений с запаздыванием. В качестве бифуркационного параметра рассматривается константа скорости активации макрофагов. Определены области в пространстве параметров модели, в частности, для скорости активации клеток врожденного иммунитета (макрофагов), при которых имеют место свойства бимультистабильности и гистерезиса, и исследованы особенности кинетики перехода между устойчивыми положениями равновесия. В целом результаты бифуркационного анализа модели ВИЧ-1 инфекции формируют теоретическую основу для разработки комбинированных иммунотерапевтических воздействий для лечения ВИЧ-1. Результаты проведенного исследования модели ВИЧ-1 инфекции для параметров процессов, отвечающих разным фенотипам динамики заболевания (типичное, длительно не прогрессирующее и быстро прогрессирующее), указывают на то, что для эффективного функционального лечения больных ВИЧ-инфекцией требуется развитие персонализированного подхода, учитывающего как свойства популяции квазивидов ВИЧ-1, так и иммунный статус пациента.

## Introduction

Human infectious disease caused by human immunodeficiency virus type 1 (HIV-1) remains a serious threat to human health worldwide, with the number of infections and deaths from as- sociated complications of the order of 1.5×106 and 0.65×106, respectively (Landovitz et al., 2023). The current approach to HIV-1 treatment involves the continued use of highly active antiretroviral therapies (Gandhi et al., 2023), which inhibit various stages of the intracellular viral reproduction cycle and thus reduce the viral load in the patient’s body. However, this approach has significant adverse side effects, as well as high treatment costs and suffers from interruption of the drug regimen (Trickey et al., 2022). For this reason, the search for therapies (Rasmussen, Søgaard, 2018; Niessl et al., 2020), including those related to the activation of immune control of virus reproduction and infection of target cells, and phy- siological mechanisms for boosting cellular homeostasis, is an urgent task (Grossman et al., 2020) that needs to be ad- dressed following a systems immunology approach (Ludewig et al., 2012, Villani et al., 2018). The research in the field of immunotherapy-based treatment of HIV-1 is at the concep- tualization stage due to the complexity of the set of processes that regulate the dynamics of infection and immune response (Landovitz et al., 2023). In this regard, the use of methods of mathematical modeling of HIV-1 infection dynamics is a tool for theoretical analysis of opportunities for viral load reduction by influencing the immune system without the use of antiviral therapy (Bocharov et al., 2022).

As has been previously noted (Bocharov et al., 2021), one of the goals of the development of mathematical models created to describe and study the dynamics of infectious diseases is the analysis of the characteristics of the dynamics sensitivity to influences of different nature, for example, in relation to perturbations of the parameters of regulatory processes or the state of the system in phase space. The results of modeling allow one to translate into a rational mode the design of com- bined control actions for correction of unfavorable infection course, in particular, from the region characterized by a high viral load to the region with a low viral load. The feasibility of the corresponding transitions is determined by the funda- mental characteristics of the modeled system – the presence of bistability and/or multistability and hysteresis. Bistability, as an ability of the system “virus–human host” to coexist in two stable steady states, justifies the search for functional cure regiments of viral infection leading to transition from a chronic stable steady state with a higher viral load to a more favor- able stable steady state with a lower viral load by inducing the activation of immune system components. The presence of the hysteresis property in bifurcation curves of a dynami- cal system makes the backstory significant, in particular, the critical importance of the branch on which the steady state of the system has been located before the subsequent change of bifurcation parameters (Khristichenko et al., 2022)

Research on mathematical modeling of HIV-1 infection dynamics in the human host has been actively developing for the last 30 years (Perelson, Nelson, 1999; Nowak, May, 2000). The key research areas were systematically presented in our earlier review (Bocharov et al., 2012). The main focus of the related papers is aimed at studying the infection kinetics during the application of antiretroviral therapy using low- dimensional models (Akın et al., 2020). Models of HIV-1 infection that consider the development of antiviral immune response are also related to the problem of estimating the infection parameters from individual patient’s data (Banks et al., 2017). Conceptual aspects of HIV-1 infection dynamics, such as multistability and hysteresis, remain an underexplored problem and the study of steady states is mainly reduced to elucidating the conditions for the existence of an infection- free equilibrium and the state of the infected organism as a function of the model parameters combined together in the basic reproductive number (Perelson, Nelson, 1999; Nowak, May, 2000).

The aim of this study is to investigate, firstly, the proper- ties of bi-, multistability and hysteresis for a model of HIV-1 infection that describes the most important blocks of virus–hu- man host interaction processes for sets of model parameters corresponding to different phenotypes of disease dynamics, i. e. known as typical progression, long-term non-progression and rapid progression courses, and, secondly, the conditions for transferring the mode of disease course from a more severe to a less severe state.

The specific objectives of this research include the bifurca- tion analysis of the model of the HIV-1 infection to identify the ranges of parameter values in which several steady states coexist, and the study of transitions between them, which are characterized by dependence on the prehistory of the state of the “virus–human host” system (hysteresis property). As a reference mathematical model for the study of stationary modes of HIV-1 infection dynamics and transitions between them, we consider a previously developed mathematical mo- del (Hadjiandreou et al., 2009), which is characterized by the following essential properties:

• it describes the entire kinetics of infectious disease from early infection to the AIDS stage, • it comprises a fairly complete spectrum of infection and immune response processes, • the model parameters corresponding to different phenotypes of infection dynamics are provided, • the description of antiretroviral therapy is included, • the antiretroviral therapy with consideration of side effects is discussed and analyzed as an optimal control problem.

Previously, we used this model to develop a more complete description of the immune response to HIV infection that takes into account neuroendocrine regulation of the immune system, in particular, the influence of hormones (TSH, T3, T4) on the immune response, and to examine an optimal antiviral therapy on its basis (Savinkova et al., 2019).

The present work consists of four sections. Section “Ma- te rials and metods” describes the considered mathematical model of HIV-1 infection and the numerical methods used to analyze the model. Section “Results” presents the results of studying the steady states of the model system by tracing them by varying the model parameters, and the analysis of steady state changes under therapeutic interventions, which are de- s cribed in the model as additional control variables on the right-hand sides of the model equations, i. e. in the terms for infection of target cells and virus replication processes. The application of the results of this work to the theoretical deve- lopment of new approaches to HIV-1 treatment is discussed in Section “Discussion”.

## Materials and methods

Let us define the basic concepts that will be used throughout the paper.

 “Functional cure of HIV-1 infection” is an approach to the- rapy of the chronic infection associated with activation of immune control of viral replication and target cell infection that allows to exclude the use of antiretroviral drugs. • “Bi-(multi)stability” is the property of a dynamical system to have two (or more) stable steady state solutions at the same parameter values. • “Hysteresis” is a property of a dynamical system that is characterized by the dependence of its steady state on the backstory curve for the parameter being varied, which can be used for transition from one steady state to another by varying the parameters.

## Mathematical model of HIV infection

The considered mathematical model of HIV infection is for- mulated in (Hadjiandreou et al., 2009) as a system of 11 or- dinary differential equations. It describes the rate of change in time of the following concentrations: wild-type (wt) virus V1; mutated virus V2; CD4+ T cells T; wt virus-infected CD4+ T cells, T1; CD4+ T cells infected with mutated T2; latently wt virus infected T cells TL1; CD4+ T cells, latently-infected with mutated virus-infected T cells TL2; macrophages M; wt virus-infected macrophages M1; macrophages infected with mutated virus M2; cytotoxic CD8+ T lymphocytes CTL. The system includes three blocks of equations: (1) the CD4+ T cell block, (2) the macrophage and CTL block, and (3) the wild-type and mutant virus block.

The first block includes the equation for CD4+ T cells:

**Formula. 1.**
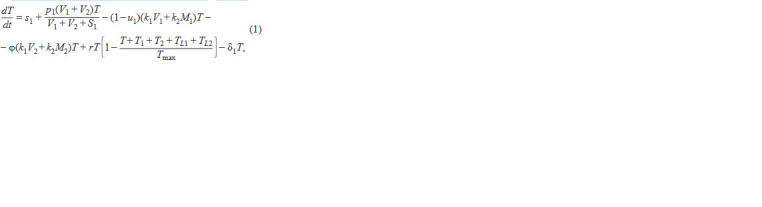
Formula. 1.

where the 1st term describes the constant influx of CD4+ T cells from the thymus, the 2nd term describes antigen- induced division, the 3rd term describes the loss due to infection by wt viruses and population of wt virus-infected macrophages, the 4th term describes the infection by mutated viruses and population of mutant virus-infected macrophages, the 5th term describes the homeostatic proliferation, and the 6th term describes natural cell death. It also includes the fol- lowing two equations for infected CD4+ T cells:

**Formula. 2.**
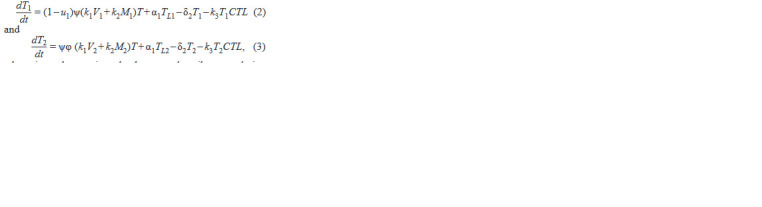
Formula. 2.

where in each equation, the 1st term describes population growth due to infections by wt or mutated virus and wt and mutated virus-infected macrophages; the 2nd term describes the transition of latently infected cells to productively infected cells; the 3rd term describes natural cell death, and the 4th term describes the CTL-mediated destruction of infected cells. The last two equations of the first block read as follows:

**Formula. 3.**
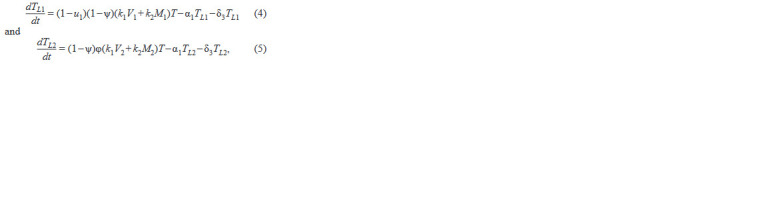
Formula. 3.

where in each of the equations the 1st term describes popula- tion growth due to infection by wt or mutated viruses and wt or mutated virus-infected macrophages; the 2nd term describes the transition of latently infected cells to productively infected cells, and the 3rd term describes natural cell death.

The second block for macrophage and CTL dynamics consists of the equation:

**Formula. 4.**
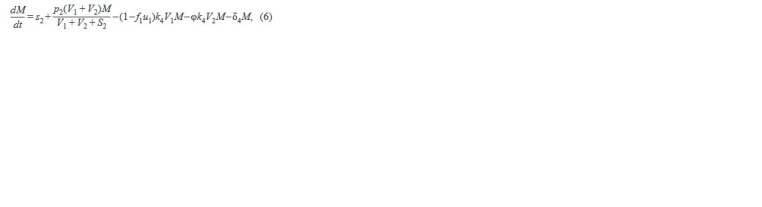
Formula. 4.

where the 1st term describes the constant influx of cells from the bone marrow, the 2nd term describes the process of activa- tion of macrophages with the possibility of their subsequent division due to chronic inflammation caused by HIV-1 infec- tion, the 3rd term describes the infection of macrophages by wt viruses, the 4th term describes infection of macrophages by mutated viruses, and the 5th term describes natural death. This block also includes two equations for infected macro- phages

**Formula. 5.**
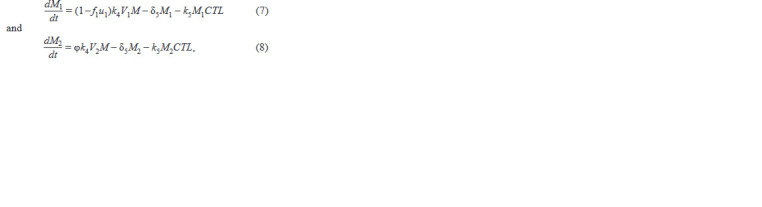
Formula. 5.

where the 1st term describes the population growth due to in- fection of macrophages by wt or mutated viruses, the 2nd term describes natural death, and the 3rd term describes destruction by CTL effect. Finally, it includes the equation:

**Formula. 6.**
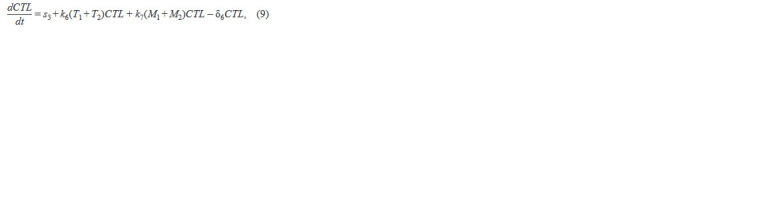
Formula. 6.

where the 1st term describes a constant influx of CD8+ T cells from the thymus, the 2nd term describes the clonal prolifera- tion induced by infected CD4+ T cells, the 3rd term describes the clonal proliferation induced by infected macrophages, and the 4th term describes cell death.

The third block of wt and mutant virus dynamics consists of two equations

**Formula. 7.**
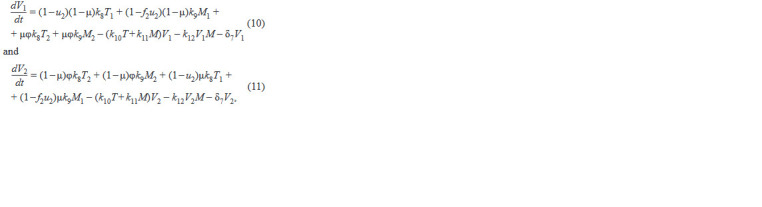
Formula. 7.

where in each of the equations the 1st term describes virus production by infected CD4+ T cells, the 2nd term describes virus production by infected macrophages, the 3rd term de- scribes virus production by infected CD4+ T cells following mutations, the 4th term describes virus production by infected macrophages following mutations, the 5th term describes virus uptake by cells when infecting target cells, the 6th term describes virus elimination by the innate system immune cells, and the 7th term describes natural virus death. The biological meaning of the system parameters and their acceptable ranges are taken from the original work (Hadjiandreou et al., 2009) and summarized in Table 1.

**Table 1.**
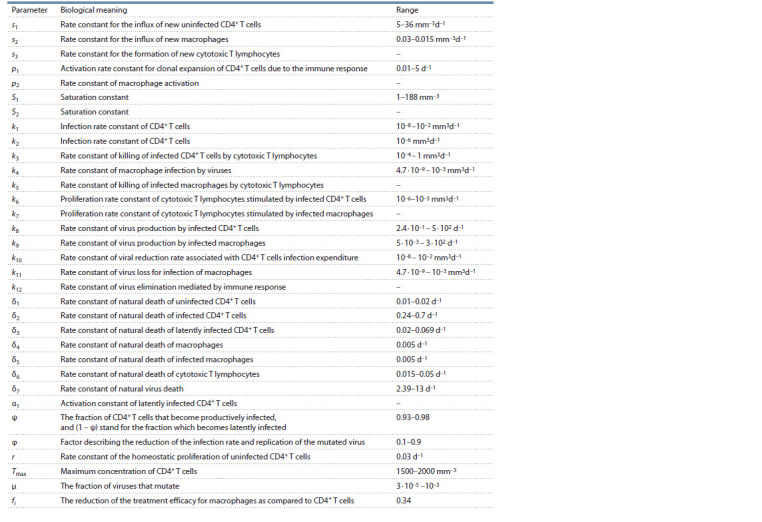
Biological meaning of the model parameters and their admissible ranges

## Optimal control problem

In the article (Hadjiandreou et al., 2009), the possibility of optimizing the mode of administration of protease (RDV) and reverse transcriptase (3TC, ZDV) inhibitors was studied. Their concentrations are described by the following equa- tions,

**Formula. 8.**
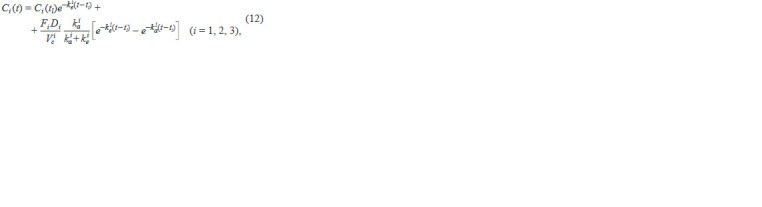
Formula. 8.

where i is the drug index, tl is the time of drug administra- tion, Di is the dose of the administered drug, Fi is the ab- solute bioavailability of the drug, k i a is the drug absorption rate, k i e = Cli /V i c is the drug elimination rate constant (Cli is the elimination rate, V i c is the drug distribution volume). The values of all the above parameters are summarized in Table 2

**Table 2.**
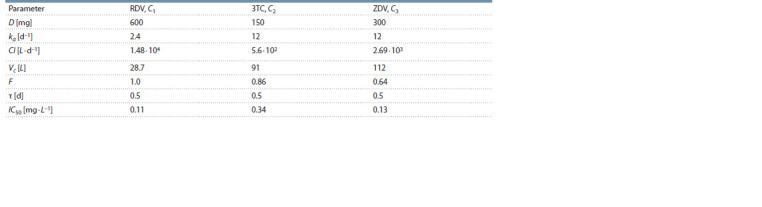
Parameter values for the pharmacokinetic equations (12)

Control variables u1 and u2 were assumed to depend on the concentration of these drugs as follows:

**Formula. 9.**
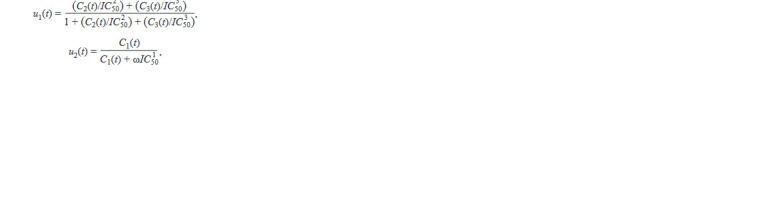
Formula. 9.

where Ci (t) is the concentration of drug i in plasma at time t, IC i 50 is the average concentration of the drug that provides 50 % inhibition of virus replication processes. The parame- ter ω is a conversion factor between the value of the average concentration of the drug providing 50 % inhibition of virus replication processes IC50 obtained in vitro, and the same value obtained in vivo. The value ω = 1 was used in the computations. The goal of optimization in the original work was to achieve the maximum concentration of CD4+ T cells (variable T in the system (1–11)) with the minimum index of adverse drug effects (Joly, Pinto, 2006

**Formula. 10.**
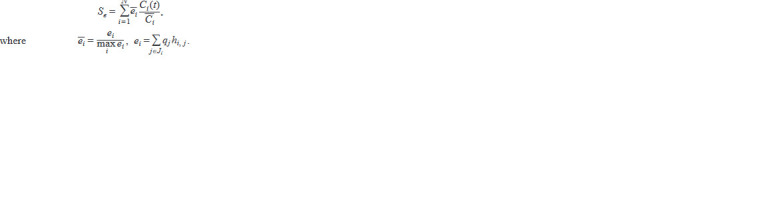
Formula. 10.

Here Ji is the set of side effects from the drug i, Ci is the average concentration of the drug i at steady state at standard dosage, that is, according to the regulation rules of antiretro- viral therapy, ei (ei) is the magnitude (normalized value) of the side effect caused by the drug i at the standard dosage, hi, j is the frequency of occurrence of the side effect j when exposed to the drug i at the standard dosage, and qj is the relative magnitude of the side effect j, that is, its “undesir- ability”.

The optimal control problem was formulated as a problem of maximizing the functional that depends on the concentra- tion of CD4+ T lymphocytes and the severity of side effects:

**Formula. 11.**
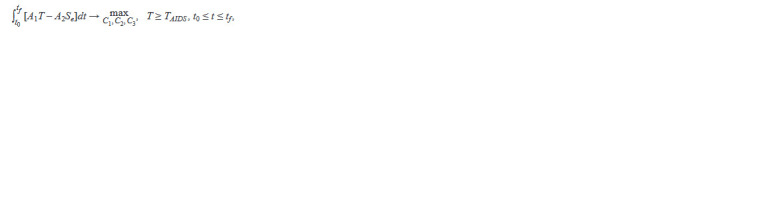
Formula. 11.

where A1 = 1 and A2 = 1000 are weight coefficients, t0 and tf specify the optimization time interval, and the condition T ≥ TAIDS prevents the cell concentration from falling below the threshold corresponding to the development of AIDS (200 cells/mm–3).

Three sets of parameter values corresponding to different phenotypic variants of HIV infection course were considered: typical progression course (TP), rapid progression course (RP) and long-term non-progression course (LNTP). The parameter values in these sets are summarized in Tables 3 and 4

**Table 3.**
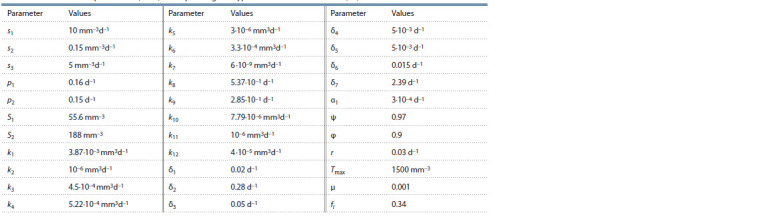
Values of model parameters (1–11) corresponding to a typical course of HIV infection (TP)

**Table 4.**
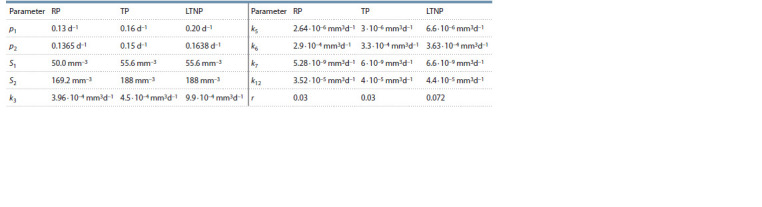
Values of model parameters (1–11) corresponding to different HIV infection phenotypes

In the original study (Hadjiandreou et al., 2009), a more effective regimen of drug administration based on optimiza- tion results was found to be superior to the standard treatment regimen for the parameters of a patient with a typical course of HIV infection with an initial CD4+ T cell concentration equal to 350 mm–3. While the standard treatment of the patient managed to keep the concentration of CD4+ T cells above the AIDS threshold for about 2,500 days, the treatment regimen based on the optimization results extended it to longer than 10,000 days with a more than four times lower value of the side-effect index Se.

## Numerical methods

To numerically integrate the system (1–11), we used an im- plicit second-order BDF2 scheme (Hairer et al., 1987) on a sufficiently fine uniform grid built in half-interval t ≥ 0. The accuracy of the results for the selected grid step was checked in all experiments requiring time integration. Symbolic com- putation methods (Geddes et al., 1992) implemented in the NSolve procedure of Mathematica were used to find steady states for given parameter values. To trace the solutions by varying parameters (i. e., to investigate the dependence of steady states of the system (1–11) on the parameters), we used the original algorithm proposed in (Nechepurenko et al., 2020). The study of asymptotic stability of a given steady state was reduced to the computation of eigenvalues of the system linearized with respect to this steady state and checking that all the found eigenvalues lie strictly in the left half-plane. To compute the eigenvalues, we used the standard QR algorithm (Golub, Van Loan, 1989)

## Results

Bifurcation analysis

This section presents the results of the study of the dependence of steady states of the model of HIV infection dynamics on the activation rate of macrophages p2 leading to their divi- sion, for three sets of values of the other parameters as given in “Materials and methods”. Earlier, for the mathematical model of hepatitis B virus infection we showed the key role of the activation rate of innate immunity in the determina- tion of different modes of hepatitis dynamics (Khristichenko et al., 2023), the analog of which in this model is p2. The parameter p2 was varied in the range from 0.13 to 0.17. The range of variation of the parameter p2 was chosen to cover those values that correspond to the kinetics of innate immunity activation for three different modes of disease course (typical progression, long-term non-progression and rapid progression) shown in Table 4.

**Fig. 1.**
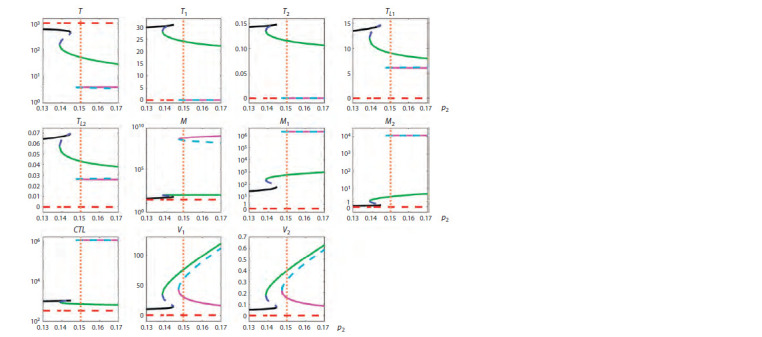
Tracing of steady states by parameter p2 for typical progression (TP) showing the presence of bistability and hysteresis. Solid lines indicate stable steady states, dashed lines indicate unstable steady states, and different colors indicate different steady states. The vertical orange dotted line indicates the value of the parameter p2 corresponding to a TP course of infection.

**Fig. 2.**
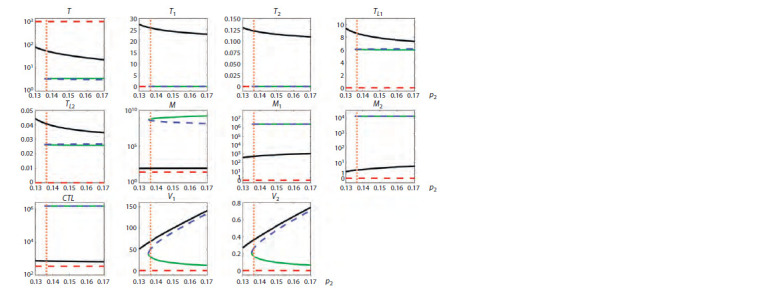
Tracing of steady states by p2 for rapid progression (RP) showing bistability. Solid lines indicate stable steady states, dashed lines indicate unstable steady states, and different colors indicate different steady states. The vertical orange dotted line indicates the value of the parameter p2 corresponding to a RP course of the infection.

**Fig. 3.**
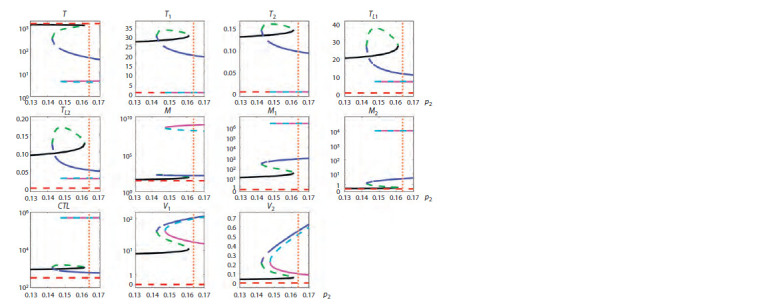
Tracing of steady states by p2 for long-term non-progression (LTNP) showing multistability Solid lines indicate stable steady states, dashed lines indicate unstable steady states, and different colors indicate different steady states. The vertical orange dotted line indicates the value of the parameter p2 corresponding to a LTNP course of the infection.

Figures 1–3 summarize the tracing results. The vertical orange dotted line indicates the value of parameter p2 taken from the corresponding parameter set, solid lines show stable steady states and dashed lines show unstable steady states, different colors indicate different steady states. It should be noted that the leading eigenvalues of the linearized equations corresponding to unstable steady states were real in all cases considered. Therefore, stable periodic solutions, which could otherwise be in the neighborhood of unstable steady states (Khristichenko, Nechepurenko, 2021), were absent in the considered cases.

Bistability. For a typical progression (TP) infection cour- se (see Fig. 1), it can be seen that bistability is present at 0.138 < p2 < 0.144 (black and green lines) and at 0.147 <p2 < < 0.17 (green and purple lines). For a rapid progression (RP) course (see Fig. 2), bistability is present at 0.135 < p2 < 0.17 (black and green lines). For a long-term non-progression (LTNP) course (see Fig. 3), bistability is present at 0.161 < p2 < < 0.17 (blue and purple lines). The presence of two different stable steady states means that there is a possibility of estab- lishment of a milder or more severe form of the disease in the same patient, depending on the patient’s backstory. Note that for a RP infection course, both equilibria are character- ized by a depleted CD4+ T cell population, with macrophages being the dominant source of viruses. For such patients, the task of treatment becomes more complicated, because it is necessary to find changes in the system parameters, at which the equilibrium with a higher level of CD4+ T cells would emerge.

In general, the obtained estimates of the areas of bistabil- ity together with the characteristics of bifurcation diagrams show that as the severity of the infection increases, i. e., as we move from long-term non-progressors to typical progressors and further to rapid progressors, the range of values of the activation rate of innate immunity cells, at which bistability takes place, increases. At the same time, some features of bifurcation diagrams change as well. These specific features of the response of an HIV-infected patient should be taken into account and used in the design of immunomodulatory regimes

Multistability. The multistability property, as shown in Figure 3, occurs in the case of a LTNP infection course at 0.146 < p2 < 0.161 (black, blue and purple lines). The respec- tive stable steady states correspond to different forms of the disease course in terms of the severity and efficacy of the im- mune response. Thus, the spectrum of possible stable steady- state modes of HIV-1 infection dynamics is more diverse in long-term non-progressors.

Hysteresis. The presence of the hysteresis property for this model is demonstrated in Figure 1. In particular, the behavior of the curves shows that if a patient belonging to typical pro- gressors was initially on the lower green branch at p2 = 0.14, then it is sufficient to reduce the value of p2 to a value slightly less than 0.138, which will cause a spontaneous transition to the state depicted by the black line, characterized by a higher T cell concentration and lower viral load. It is then possible to increase the value of the parameter p2 to the original value while staying on the same black line

Hysteresis also occurs for parameters corresponding to the LTNP infection course, as demonstrated in Figure 3. The state depicted by the blue line at p2 = 0.155 is stable, but it loses stability at p2 smaller than 0.146. With further reduc- tion of the parameter value, the system will move from a less favorable state (green branch) to a stable state with a higher concentration of CD4+ T cells and lower viral load, depicted by the black solid line. After that, it is possible to return to the initial value of the parameter while remaining on this stable steady state branch.

**Fig. 4.**
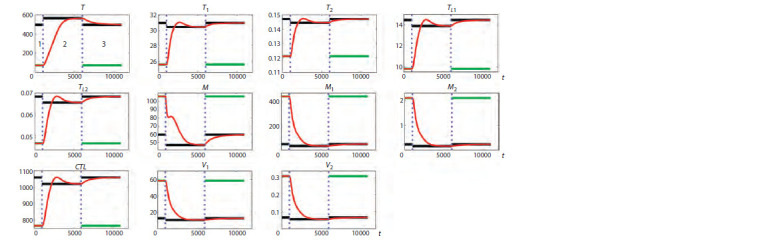
Demonstration of the transition kinetics from a less favorable steady state to a more favorable steady state in the presence of hysteresis for typical progression (TP), where p2 = 0.143 in regions 1 and 3, and p2 = 0.136 in region 2. The horizontal axis indicates time in days. The red solid line shows the dynamics of the model variables, the blue vertical dotted lines show the partitioning into regions 1–3, and the horizontal solid lines show the stable steady states of the variables in these regions.

Of practical importance is the question of the kinetics of the transition between different steady states when utilizing the hysteresis property. For a TP disease course, Figure 4 shows the transition dynamics from a less favorable state to a more favorable state for a system with hysteresis. It takes about 5,000 days to realize this transition with constant values of other system parameters. These results justify the relevance of further detailed study of such transitions.

Changes in steady states with a single administration of drugs

**Fig. 5.**
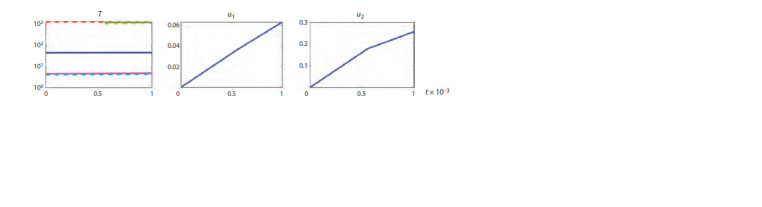
Time dependencies t (in days) of the steady state variable T and control variables u1(t) and u2(t) at 0 ≤ t ≤ 0.001 for long-term non-progression (LTNP). Solid lines on the graph T(t) correspond to stable steady states, dashed lines – to unstable ones.

It is of independent interest to understand how the steady states of a system change under optimal control (Hadjiandreou et al., 2009). To this end, we investigated the time dependence of equilibria under therapeutic interventions u1(t), u2(t), which enter the right-hand sides of the model equations in the terms for the processes describing the infection of target cells and virus replication. Figure 5 shows the appearance of two new steady states at t > 0.0005, i. e., a change in the structure of the phase space of the model.

**Fig. 6.**
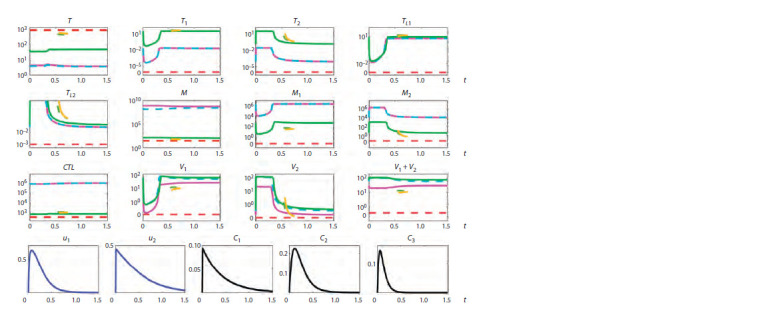
Steady states and control actions for typical progression (TP) infection course. Solid lines indicate stable steady states, dashed lines indicate unstable steady states, different colors indicate different steady states. The horizontal axis shows time in days.

**Fig. 7.**
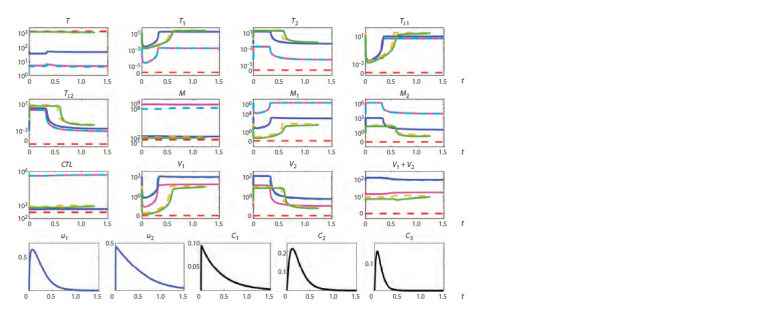
Steady states of the model and control actions for long-term non-progressive flow (LTNP). Solid lines indicate stable steady states, dashed lines indicate unstable steady states, different colors indicate different steady states. The horizontal axis indicates time in days.

**Fig. 8.**
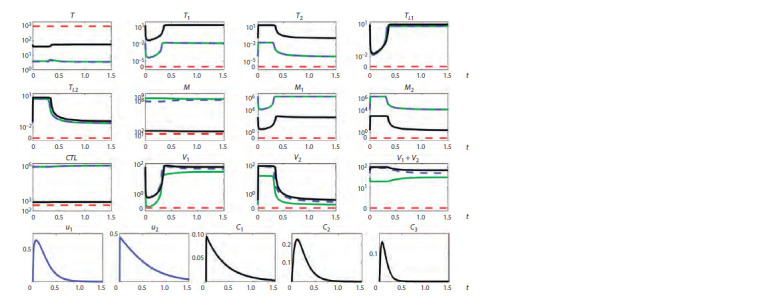
Steady states of the model and control variables for rapid progression (RP) infection course Solid lines indicate stable steady states, dashed lines indicate unstable steady states, and different colors indicate different steady states. The horizontal axis indicates time in days.

Figures 6–8 illustrate the steady-state changes when RDV, 3TC, and ZDV drugs are administered, the effects of which are modeled using functions C1(t), C2(t), C3(t) through the control variables u1 and u2. The drugs are administered once at time t = 0. The solid lines indicate stable steady states and the dashed lines indicate unstable states, different colors in- dicate different steady states. The numerical results indicate that as the values of the control variables change, both stable and unstable steady states appear and then disappear. Thus, the application of optimal control methods leads to a change in the structure of the phase space of the model.

For all three variants of the course of HIV-1 infection, for one branch of the steady-state solutions, there is a short-term decrease in the values of variables characterizing the number of CD4+ T cells and an increase in viral load due to an in- crease in the number of mutants and a decrease in steady-state concentrations of wild-type viruses. On the second stable branch, an opposite process takes place. In this case, in the case of a long-term non-progression course of HIV-1, the third branch of the stable equilibrium appears, which is characte- rized by a low viral load and, therefore, corresponds to more favorable dynamics. Thus, the impact of optimal control on the characteristics of equilibrium states depends essentially on the disease course phenotype (model parameters) and the neighborhood of the equilibrium in which the patient is in the case of bistability.

Thus, the response to the perturbation of the right-hand sides of the equations is qualitatively the same. The structure of the phase space changes, and as the control function impact is weakened, both stable and unstable steady states emerge and then disappear.

## Discussion

A stable coexistence of the HIV-1 population and immune processes in the human body in various quantitative ratios is fundamentally important for the development of new strate- gies of HIV-1 therapy that belong to the category of func- tional treatment (cure) (Bocharov et al., 2022). In essence, it is the possibility of transferring the “virus–human host” system from a clinically more severe state to a milder infec- tion stable steady state due to activation of immune defense mechanisms without further use of antiretroviral drugs that block viral replication. The presence of bi- or multistability indicates that by perturbing a certain trajectory of the system in the phase space, the transfer of the infectious disease to a more favorable regime can be accomplished. Both clas- sical optimal control methods (Hadjiandreou et al., 2009; Bocharov et al., 2015) and our previously proposed methods based on optimal disturbances (Nechepurenko, Khristichen- ko, 2019; Khristichenko, Nechepurenko, 2022) exist as tools for constructing an appropriate control. Furthermore, there could be a case when a change in the kinetic parameters of biological and physiological processes is required to move the system into the region of bi- or multistability. The presence of hysteresis allows one to develop treatment approaches that utilize temporary parametric shifts with subsequent return to the initial values of the changed parameters. The identified properties of the mathematical model of HIV-1 infection, which has a fairly typical structure, theoretically confirm the potential feasibility of corresponding combination immune- based therapeutic interventions (Landovitz et al., 2023).

The obtained estimates of the parameter regions enabling the existence of bistability together with the characteristics of bifurcation diagrams show that as the severity of the HIV-1 infection increases, i. e. in the transition from long-term non- progressor to typical progressor and further to rapid progres- sor phenotype, the range of values of the activation rate of innate immunity cells, at which the bistability takes place, increases. Meanwhile, the properties of bifurcation diagrams also change. These specific features of the response of an HIV-infected patient should be taken into account and used in the design of immunomodulatory regiments.

Finally, we showed that the impact of optimal control on the characteristics of equilibria depends significantly on the phenotype of HIV-1 infection (determined by system param- eters) and the neighborhood of the equilibrium in which the patient is located in the case of bi- or multistability.

## Conclusion

In this paper, we have computed and numerically analyzed the steady states of the mathematical model of HIV-1 infection for sets of parameters corresponding to phenotypically different variants of the course of the infection: typical progression, long-term non-progression and rapid progression. The re- sults of the bifurcation analysis of the HIV-1 infection model indicate that implementation of an effective functional cure of infected patients requires the development of a personal- ized approach that takes into account both the properties of the HIV-1 quasispecies population and the patient’s immune status. Overall, our study forms a theoretical basis for the development of combination immune-based therapy of HIV-1 infected patients.

## Conflict of interest

The authors declare no conflict of interest.
